# Healthcare Resource Utilization and Costs Among Commercially Insured Patients With Advanced or Recurrent Endometrial Cancer Initiating First-Line Therapy in the United States

**DOI:** 10.36469/001c.88419

**Published:** 2023-11-08

**Authors:** Monica Kobayashi, Jamie Garside, Joehl Nguyen

**Affiliations:** 1 Real-World Analytics GSK, Durham, North Carolina, USA; 2 Value Evidence and Outcomes (Oncology) GSK, London, UK; 3 Real-World Analytics GSK

**Keywords:** Advanced/recurrent endometrial cancer, healthcare resource utilization, first-line treatment, cost outcomes, US database study, metastatic disease

## Abstract

**Background:** Endometrial cancer (EC) represents a substantial economic burden for patients in the United States. Patients with advanced or recurrent EC have a much poorer prognosis than patients with early-stage EC. Data on healthcare resource utilization (HCRU) and costs for patients with advanced or recurrent EC specifically are lacking.

**Objectives:** To describe HCRU and costs associated with first-line (1L) therapy for commercially insured patients with advanced or recurrent EC in the United States.

**Methods:** This was a retrospective cohort study of adult patients with advanced or recurrent EC using the MarketScan® database. Treatment characteristics, HCRU, and costs were assessed from the first claim in the patient record for 1L therapy for advanced or recurrent EC (index) until initiation of a new anti-cancer therapy, disenrollment from the database, or the end of data availability. Baseline demographics were determined during the 12 months before the patient’s index date.

**Results:** A total of 7932 patients were eligible for inclusion. Overall, mean age at index was 61 years, most patients (77.3%) had received prior surgery for EC, and the most common 1L regimen was carboplatin/paclitaxel (59.1%). During the observation period, most patients had at least one healthcare visit (all-cause, 99.9%; EC-related, 82.8%), most commonly outpatient visits (all-cause, 91.4%; EC-related, 68.7%). The highest mean (SD) costs (US dollars) were for inpatient hospitalization for both all-cause and EC-related events (8396[15,130] and 9436[16,784], respectively). Total costs were higher for patients with a diagnosis of metastasis at baseline than for those without a diagnosis of metastasis.

**Discussion:** For patients with advanced or recurrent EC in the United States, 1L therapy is associated with considerable HCRU and economic burden. They are particularly high for patients with metastatic disease.

**Conclusions:** This study highlights the need for new cost-effective treatments for patients with newly diagnosed advanced or recurrent EC.

## BACKGROUND

Endometrial cancer (EC) is the most common gynecological cancer in developed countries.^1^ It is most frequently diagnosed in patients 55 to 64 years of age and is often found at an early stage (66%) due to the occurrence of abnormal uterine bleeding.^2,3^ An estimated 65,950 new cases and 12,550 deaths occurred in the United States in 2022.^2,3^ In contrast to many other tumor types, EC incidence and mortality rates have risen since the mid-1990s; between 2015 and 2019, mortality rates increased 1% annually.^2^ Disease stage and histological subtype are important determinants of disease recurrence and death (eg, clear cell or serous carcinomas are more aggressive than other histologies).^4^ Despite treatment (surgery and, if needed, adjuvant therapy), disease recurrence has been reported in approximately 7% of Stage I patients and 67% of Stage IV patients^4,5^; most recurrences occur within 3 years of primary treatment.^6^ For patients with advanced or recurrent EC, the prognosis is particularly poor; the reported 5-year relative survival rate is between 18% and 69% for patients with advanced disease (depending on whether disease spread is distant or regional) and 20% for patients with recurrent EC, compared with 95% for those with early-stage disease.^2,4^

First-line (1L) treatments recommended for EC by the National Comprehensive Cancer Network® (NCCN®) are dependent on disease stage; for early-stage EC, 1L treatment recommendations include surgery and radiation therapy, whereas for advanced or recurrent EC, 1L recommendations include systemic therapy, with or without radiotherapy; the preferred systemic therapy regimen is carboplatin/paclitaxel.^7^ A recent study showed that patients in the United States who received systemic therapy incurred considerable healthcare resource utilization (HCRU) and healthcare costs.^8^ That study included both patients with early-stage and locally advanced disease; however, patients with advanced or recurrent EC may have different HCRU and economic burdens than those with early-stage disease, due to the differences in treatment patterns and outcomes.^2,4,7^ Data on HCRU and costs for these patients specifically are lacking.

The primary objective of the current study was to describe the HCRU and costs associated with 1L therapy among patients with advanced (de novo Stage III or IV disease) or recurrent EC included in the US-based MarketScan® database. The secondary objectives were to describe baseline demographics and clinical and treatment characteristics among patients with advanced or recurrent EC, both overall and stratified by metastasis at baseline and radiation therapy.

## METHODS

### Data Source and Study Design

This was a retrospective cohort study of adult female patients with EC, using the IBM MarketScan® Commercial Claims and Encounters and Medicare Supplemental databases (referred to as the MarketScan® database). The MarketScan® database collects de-identified medical, drug, and dental claims from more than 245 million privately and publicly insured patients. Variables were assessed based on administrative codes for clinical diagnoses, medications, and medical procedures that were identified from healthcare claims in the MarketScan® database. Diagnoses were coded using the *International Classification of Diseases, Ninth and Tenth Revision, Clinical Modification* (ICD-9-CM and ICD-10-CM).

**Figure 1.**
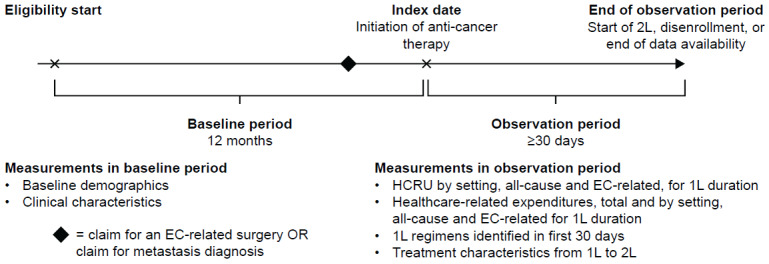
Study Design Abbreviations: 1L, first-line; 2L, second-line; EC, endometrial cancer; HCRU, healthcare resource utilization.

The study design is shown in **Figure 1**. The baseline period was defined as the 12-month period between the start of a patient’s eligibility period and the patient’s index date, during which inclusion criteria, exclusion criteria, and baseline characteristics were assessed. Although patients could have more than 12 months of continuous enrollment before the index date, a 12-month fixed period was used to assess baseline characteristics. The index date was defined as the first claim in the patient record with an anti-cancer treatment recommended as 1L therapy for advanced or recurrent EC by the NCCN® Clinical Practice Guidelines in Oncology (NCCN Guidelines®).^7^ Anti-cancer medications identified in the 30-day period after and including the index date were defined as part of 1L therapy. The observation period to assess HCRU and cost outcomes associated with 1L therapy was defined as the period from the index date until either the start of a new anti-cancer therapy, disenrollment from the database, or the end of data availability, whichever occurred first. For patients who initiated a new anti-cancer therapy, the end of 1L therapy was defined as the day before the initiation of treatment not part of the 1L regimen at the class level (second-line [2L] therapy) or resumption of the same treatment regimen after an allowable gap of more than 180 days from the last administration (accounting for re-treatment). 1L therapy was permitted to include multiple claims of treatment with the same agent (if there was no gap of more than 180 days between administration dates) and treatment within the same medication class (eg, platinum-based therapies).

### Study Population

Staging and recurrence information were not available in the MarketScan® database; therefore, EC surgery or diagnoses of metastasis were used to identify a cohort with the characteristics of an advanced or recurrent EC population. Patients with advanced or recurrent disease were identified based on a metastatic code (indicating Stage IV) or whether they had history of surgery for EC (hysterectomy and/or salpingo-oophorectomy) and had initiated anti-cancer medications recommended for 1L treatment of Stage III/IV EC. Patients were required to be ≥18 years of age at index, to be continuously enrolled in a health plan with medical and pharmacy benefits for ≥12 months before and ≥30 days after the index date, and to have ≥2 claims with diagnosis codes for EC (ICD-9-CM 179.x and 182.x; ICD-10-CM C54 and C55 [relating to EC]) and ≥1 claim either for surgery for EC or with a metastatic code (ICD-9-CM codes 196–199; ICD-10-CM codes C77–C80, C7B [relating to metastasis]) ≤12 months before and including the index date (**Supplementary Table S1**). Patients were also required to have ≥1 claim in the 1L setting (the first claim defined as the index date) between 2011 and 2020. To ensure that the first claim corresponded to 1L therapy initiation, patients were excluded if they had ≥1 claim for an anti-cancer therapy (including hormonal therapies) ≤12 months before (not including) the index date or ≥1 claim for pelvic radiation in the same period.

### Study Assessments

Baseline demographic and clinical characteristic data were evaluated during the 12-month period preceding or on the index date and were reported for all patients (overall cohort) and stratified by radiation therapy and metastasis at baseline. Demographic and clinical characteristics included age, US region, year of index, payor, comorbidities (using the Klabunde adaptation of the Charlson Comorbidity Index [CCI]),^9–11^ and EC surgery type. Treatment outcomes included type of 1L regimen (including medication class), duration of 1L therapy (index date to the end of 1L therapy), and duration of follow-up (index date to the earliest date from the day before the start of 2L therapy, disenrollment from the database, or the end of data availability).

HCRU and cost outcomes were determined across the observation period and were categorized as all-cause or EC-related. EC-related HCRU and costs were defined as claims associated with a primary diagnosis code for EC. The number of patient visits/medication administrations and costs (US dollars in 2020, per patient per month [PPPM]) were assessed across all settings (medication, emergency department [ED], inpatient, outpatient, office/clinic, skilled nursing facility, other locations) and for all settings other than medication administration/costs (referred to as non-pharmacy medical encounters).

All study outcomes were reported for the overall cohort and stratified by whether the patient met the inclusion criteria based on a metastasis or surgery (no metastasis) claim as well as whether they received radiation therapy in the 1L setting. Costs were reported for patients with at least 1 encounter of interest (referred to as “conditional” costs).

### Data Analysis

Descriptive statistics are presented for all demographic, clinical, and treatment characteristics data. The Statistical Analysis System (SAS) Studio version 3.81 or the latest version (SAS Institute, Cary, North Carolina), was used to identify the cohort and assess treatment characteristics, and the Panalgo Instant Health Data tool was used to analyze HCRU/cost outcomes and to generate report tables. To account for different lengths of observation periods among study patients, HCRU and costs were combined for the duration of follow-up and calculated as PPPM (defined for each patient as the sum of all costs during the follow-up period divided by the number of follow-up months), an approach commonly used in non-experimental study settings. For medication HCRU and costs, both medication fills and administration of therapies (in an outpatient setting) were considered and adjusted for inflation to 2020 US dollars. Means (SDs) or medians (interquartile ranges [IQRs]) were reported for continuous variables as appropriate, whereas counts and percentages were reported for categorical variables. Cost data were adjusted for 2023 inflation according to the Consumer Price Index (CPI) series.^12^

## RESULTS

### Study Population

**Figure 2.**
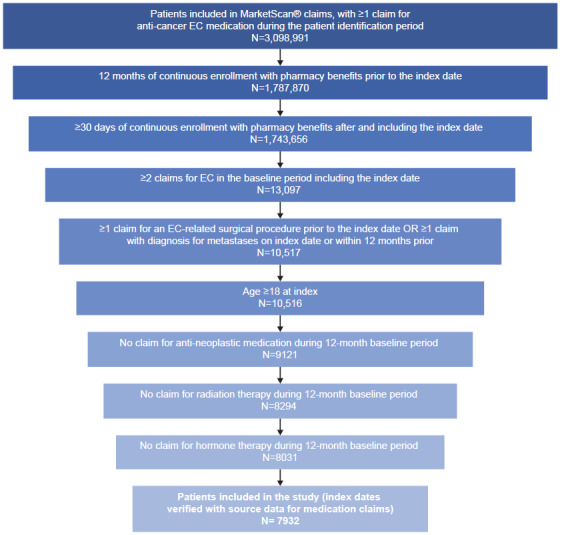
Cohort Attrition Abbreviation: EC, endometrial cancer.

A total of 7932 patients from the MarketScan® database were eligible for inclusion (**Figure 2**). Of these, 5287 patients (66.7%) had metastasis at baseline and 2233 (28.2%) had received prior radiation therapy in the 1L setting.

### Baseline Characteristics

In the overall cohort, the mean age at index was 61 years, the mean (SD) non-cancer CCI was 4.68 (3.16), and most patients (n = 6132, 77.3%) had undergone prior surgery for EC (**Table 1**). Patient characteristics were broadly similar between patients with no metastasis at baseline (**Table 1**); however, compared with patients with metastasis at baseline, patients without had a lower mean CCI (0.6 vs 6.72), and a smaller proportion had received radiation therapy in the 1L setting (n = 1228, 23.2% vs n = 1005, 38.0%). All patients without metastasis at baseline had undergone surgery for EC as an artifact of the inclusion criteria. Baseline characteristics by radiation subgroup are shown in **Supplementary Table S2.**

**Table 1.** Baseline Patient Characteristics

**Patient Characteristics**	**Overall Cohort** **(N = 7932)**	**Metastasis at Baseline (n = 5287)**	**No Metastasis at Baseline (n = 2645)**
**Age at index (y), mean (SD)**	61.0 (10.1)	61.1 (10.2)	60.7 (10.1)
**US Census region, n (%)**			
**Midwest**	2028 (26.5)	1418 (27.8)	610 (23.9)
**Northeast**	1691 (22.1)	1179 (23.1)	512 (20.1)
**South**	2701 (35.3)	1690 (33.2)	1011 (39.7)
**West**	1224 (16.0)	809 (15.9)	415 (16.3)
**NR**	288 (3.6)	191 (3.4)	97 (3.7)
**Non-cancer CCI, mean (SD)**	4.68 (3.2)	6.72 (1.4)	0.6 (1.0)
**No. of non-⁠cancer CCI conditions, n (%)**			
**0**	4829 (60.9)	3083 (58.3)	1746 (66.0)
**1–2**	2447 (30.9)	1708 (32.3)	739 (27.9)
**≥3**	656 (8.3)	496 (9.4)	160 (6.1)
**Payor, n (%)**			
**Commercial**	5830 (73.5)	3861 (73.0)	1969 (74.4)
**Medicare**	2102 (26.5)	1426 (27.0)	676 (25.6)
**History of EC surgery, n (%)^a^**			
**No EC surgery**	1800 (22.7)	1800 (34.1)	0
**Subtotal hysterectomy**	94 (1.2)	60 (1.1)	34 (1.3)
**Total hysterectomy**	4438 (56.0)	2442 (46.2)	1996 (75.5)
**Radical hysterectomy**	2306 (29.1)	1315 (24.9)	991 (37.5)
**Salpingo-oophorectomy**	2418 (30.5)	1499 (28.4)	919 (34.7)
**Bilateral salpingo-oophorectomy**	879 (11.1)	686 (13.0)	193 (7.3)
**Hysterectomy, NOS**	1892 (23.9)	1134 (21.5)	758 (28.7)
**Radiation therapy in 1L, n (%)^a^**	**2233 (28.2)**	**1228 (23.2)**	**1005 (38.0)**
**EBRT**	819 (10.3)	573 (10.8)	246 (9.3)
**Brachytherapy**	1768 (22.3)	884 (16.7)	884 (33.4)

^a^Surgery and radiation categories are not mutually exclusive.

Abbreviations: 1L, first-line; CCI, Charlson Comorbidity Index; EBRT, external beam radiotherapy; EC, endometrial cancer; NOS, not otherwise specified; NR, not reported.

### Treatment Characteristics and Duration of Therapy

**Figure 3.**
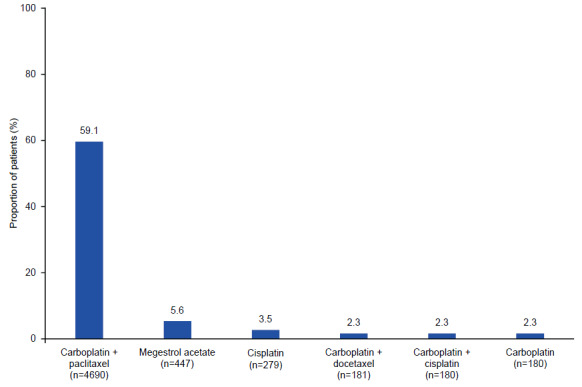
Top 6 Treatment Regimens in the Overall Cohort

In the overall cohort, the most common 1L treatment regimen was carboplatin/paclitaxel (n = 4690, 59.1%); except for megestrol acetate (n = 447, 5.6%), all other treatment regimens were used in fewer than 5% of cases (**Figure 3**). The most common 1L treatment class was platinum combination therapy (n = 5790, 73.0%; **Supplementary Figure S1A**). Carboplatin/paclitaxel and platinum combination therapy were also the most common 1L treatment regimens and class for patients both with and without metastasis (**Supplementary Figure S1**). The median (IQR) duration of 1L therapy for the overall cohort was 9.4 (7.3–11.0) months; the median (IQR) duration of 1L therapy was similar for patients with and without metastasis (9.4 [7.3–11.4] and 9.4 [7.8–10.5] months, respectively). For the overall cohort, the mean (SD) duration of follow-up was 22.6 (20.3) months; the mean (SD) duration of follow-up was longer for patients without metastasis (26.8 [22.7] months) compared with patients with metastasis (20.5 [18.7] months).

### HCRU and Costs

In the overall cohort, most patients had at least one visit to a non-pharmacy medical encounter setting for both all-cause (n = 7925 [99.9%]) and EC-related (n = 6568 [82.8%]) events (**Table 2**), with a mean (SD) number of visits PPPM of 2.8 (2.6). The setting with the highest number of patients visiting at least once for both all-cause and EC-related events was outpatient visits (n=7252 [91.4%] and n=5453 [68.7%], respectively).

**Table 2.** All-Cause and EC-related HCRU and Conditional Costs by Setting

**Setting**	**All-Cause**		**EC-related**	
	**Patients, n (% of Total)**	**Total Payment PPPM in USD, Mean (SD)**	**Patients, n (% of Total)**	**Total Payment PPPM in USD, Mean (SD)**
**Non-⁠pharmacy medical encounter^a^**	7925 (99.9)	8280 (12,282)	6568 (82.8)	5245 (9606)
ED visit	2699 (34.0)	847 (1915)	408 (5.1)	961 (1413)
Inpatient hospitalization	2135 (26.9)	8396 (15,130)	1010 (12.7)	9436 (16,784)
Outpatient visit	7252 (91.4)	4793 (7489)	5453 (68.7)	3468 (5841)
**Medication fill**	7932 (100.0)	3435 (6138)	7932 (100.0)	1081 (3670)
**Total monthly cost**	**7932 (100.0)**	**11,707 (14,031)**	**7932 (100.0)**	**5425 (9673)**
Metastasis at baseline	5287 (66.7)	13,323 (15,318)	5287 (66.7)	6143 (10,958)
No metastasis at baseline	2645 (33.3)	8479 (10,286)	2645 (33.3)	3988 (6124)

^a^See **Supplementary Table S3** for median (IQR) PPPM, **Supplementary Table S4** for costs in additional non-pharmacy medical encounter settings, and **Supplementary Table S6** for costs adjusted to 2023.

Abbreviations: EC, endometrial cancer; ED, emergency department; HCRU, healthcare resource utilization; IQR, interquartile range; PPPM, per patient per month; USD, US dollars.

Overall, for non-pharmacy medical encounters, the mean (SD) all-cause cost and EC-related costs were $8280 ($12,282) PPPM and $5245 ($9606) PPPM, respectively (**Table 2**; median [IQR] PPPM reported in **Supplementary Table S3**). Across all settings, the highest mean (SD) costs were for inpatient hospitalizations for both all-cause and EC-related events ($8396 [$15,130] and $9436 [$16,784], respectively) (**Table 2; Supplementary Table S4**). Of note, the mean medication fill cost for all-cause events was approximately 3 times higher than for EC-related events (mean [SD]: $3435 [$6138] vs $1081 [$3670]). The mean (SD) total monthly costs were $11,707 ($14,031) for all-cause and $5425 ($9673) for EC-related events. All-cause costs were higher for patients with metastasis at baseline compared with those with no metastasis (**Supplementary Table S5**). For patients with metastasis, the highest mean (SD) non-pharmacy medical encounter all-cause costs were for inpatient hospitalizations ($9210 [$15,854] PPPM). A greater number of patients with ED visits and inpatient hospitalization contributed to higher overall non-pharmacy medical encounter all-cause costs for patients with metastasis compared with those with no metastasis (**Supplementary Table S5**).

All-cause and EC-related cost data adjusted to USD in 2023 are shown in **Supplementary Tables S6–S9**; for total monthly costs this would correspond to an increase from $11,707 to $12,375 for all-cause and $5425 to $5734 for EC-related costs.

## DISCUSSION

This is one of the first studies to provide insight into the clinical and treatment characteristics, costs, and HCRU of patients with advanced or recurrent EC in a commercially insured population. This study showed that US patients with advanced or recurrent EC have considerable HCRU and economic burden, supporting findings from other studies using administrative claims data, including data derived from the Optum Clinformatics Data Mart database, to assess HCRU and cost burden across all patients with EC.^8,13^

The data presented here indicate that most patients in the overall cohort (59.1%) received carboplatin and paclitaxel as their 1L treatment regimen, with platinum combination therapy being the most common treatment class (73.0%). These findings are comparable to the previous findings for patients with EC on 1L therapy, in which carboplatin and paclitaxel were the most widely used 1L therapies among patients treated with systemic therapy only.^8,13^ A recent study that also used the MarketScan® database reported that platinum-taxane combinations were the most common regimens for 1L treatment of recurrent EC after a hysterectomy between 2011 and 2019 (46.8%), with carboplatin and paclitaxel among the most commonly used agents (52.4% and 47.8% of patients, respectively).^14^

Although all-cause costs reported in this study ($11,707 PPPM) were similar to those previously reported for 1L therapy in patients with EC ($11,363 PPPM), mean costs for all-cause ED visits and inpatient hospitalizations were higher in the current study than those previously reported for patients who received 1L therapy ($847 and $8396 vs $169 and $2295, respectively). Mean all-cause outpatient costs were also lower than previously reported in some studies ($4793 vs $8380).^8^ Another study assessing cost associated with each phase of care for patients with newly diagnosed EC reported that mean PPPM adjusted costs varied depending on whether the patient was in the prediagnosis ($4350), initial ($9570), continuation ($2165), or terminal ($11,732) phase of their disease; similar variation was also observed across inpatient, ED, and outpatient settings.^15^ While this study population included patients with characteristics of advanced or recurrent disease, the lower mean costs observed in the study could be attributed to confounding factors such as disease stage, which was not directly captured in the database. HCRU reported here is generally lower than previously reported by other US studies. The proportion of patients with at least 1 all-cause ED, inpatient, and outpatient visit in this study (34.0%, 26.9%, and 91.4%, respectively) was lower than that previously reported for patients who received 1L therapy and were either newly initiated on systemic therapy (39.0%, 42.5%, and 99.8%, respectively) or newly diagnosed (42.7%, 31.5%, and 99.9%, respectively).^8,13^ These differences may reflect that the current study included patients with advanced or recurrent EC (as opposed to early-stage EC in the previously published study) as well as differences in study design. Patients in the current study also had a longer baseline period compared with the patients in the previous study, which may affect the classification of patients as ‘advanced/recurrent’ based on surgical history, and the surgery-related inclusion criteria differed substantially between the reports.^8,13^ Additionally, patients were predominantly insured by Medicare in the previously published studies,^8,13^ whereas most patients in the current study had commercial insurance. While the latest year in the study period was 2020, to provide current context given the rise in inflation over recent years, cost data were also adjusted to 2023. Based on this adjustment, all-cause monthly costs would be $12,375 and EC-related costs would be $5734 in 2023.

The current study also showed that costs were higher for patients with metastasis at baseline vs those without, highlighting the substantial burden associated with advanced or recurrent EC. Despite patients receiving chemotherapy regimens recommended and commonly used for advanced/metastatic disease, such as carboplatin and paclitaxel, long-term survival benefit for these patients remains modest.^7,16–19^ A real-world study assessing treatment patterns and outcomes in US patients with advanced/recurrent EC initiating 1L therapy based on the Flatiron Health database reported similar findings of carboplatin plus paclitaxel being the most common 1L regimen in advanced/recurrent EC. Limited long-term survival in patients receiving these therapies was demonstrated in the 1L and 2L setting, represented by time to next treatment (TTNT), which is considered a surrogate of progression-free survival (10.6 and 8.7 months, respectively).^20^ Previous studies using the MarketScan® database have documented that an EC diagnosis is associated with employment disruption^21,22^; further research should investigate the relationship between the direct economic burden of the disease and indirect components of economic burden such as potential loss of employment for the patient or family members with caring responsibilities. There is an opportunity for novel therapies to reduce the economic burden of EC, which would be welcomed by payors and patients alike.

Several limitations of this study should be considered when interpreting the results. As mentioned previously, data on clinically confirmed staging and recurrent disease were not available in the administrative claims data for MarketScan®, so it was not possible to differentiate patients by stage. Therefore, patients included in this study could have developed metastasis from a non-EC primary tumor, whereas others may have had earlier-stage EC. However, by defining 1L therapy start and duration, the study population closely resembled patients with primary advanced or recurrent disease. Left censoring bias is also acknowledged as a limitation when defining index dates using the first claim for 1L therapy; however, incorporation of a 12-month baseline period used in this study minimizes bias. In addition, in line with other observational studies, these data are retrospective in nature and thus may be prone to inaccuracies (eg, for diagnosis, procedure, and drug codes); death was also not recorded in the database and therefore could not be used directly as a criterion for the end of observation in these analyses. Furthermore, given that the results reported are from US patients based on a single medical database, caution is advised when generalizing the results to other patients with EC, including uninsured patients, patients who are not treated with systemic therapies, or patients in other countries. Despite these limitations, this study provides a valuable insight into patients with EC and the impact of EC on HCRU and economic burden in the United States.

## CONCLUSIONS

This study provides an indirect assessment of the HCRU, costs, and treatment characteristics associated with 1L therapy among patients with advanced or recurrent EC. The study showed that advanced or recurrent EC represents a high economic burden in the United States, with higher costs attributed to patients with metastasis than those with no metastasis. Despite treatment with commonly used chemotherapy regimens, patients with metastatic disease often have unfavorable outcomes. Further research is needed to investigate new cost-effective treatments that could improve outcomes for patients with newly diagnosed advanced or recurrent EC.

### Author Contributions

M.K. contributed to the concept and design of the study, participated in data acquisition and data analysis, helped with drafting of the manuscript, and performed critical revision of the paper for important intellectual content. J.G. contributed to the concept and design of the study, participated in data acquisition and data analysis, helped with drafting of the manuscript, and performed critical revision of the paper for important intellectual content. J.N. contributed to the concept and design of the study, participated in data acquisition and data analysis, helped with drafting of the manuscript, and performed critical revision of the paper for important intellectual content. All authors read and approved the final version.

### Disclosures

J.G. is an employee of GSK; M.K. and J.N. are employees of GSK and hold stocks and shares in the company.

### Data Sharing Statement

GSK makes available anonymized individual participant data and associated documents from interventional clinical studies that evaluate medicines, upon approval of proposals submitted to https://www.gsk-studyregister.com/en/. To access data for other types of GSK-sponsored research, for study documents without patient-level data, and for clinical studies not listed, please submit an enquiry via the website.

## Supplementary Material

Online Supplementary Material
