# Supplementary material for: Healthcare Resource Utilization and Costs Among Commercially Insured Patients With Advanced or Recurrent Endometrial Cancer Initiating First-Line Therapy in the United States
Source: J Health Econ Outcomes Res. 2023 Nov 8;10(2):104–10. doi: 10.36469/001c.88419 (PMC10637624; doi:10.36469/001c.88419)
Supplement: Online Supplementary Material [file jheor_2023_10_2_88419_186445.pdf]

Journal of Health Economics and Outcomes Research

Online Supplementary Material

Healthcare Resource Utilization and Costs Among Commercially Insured Patients With Advanced or Recurrent Endometrial Cancer Initiating First-Line Therapy in the United States. *JHEOR*. 2023;10(2):104-110. doi:10.36469/jheor.2023.88419

Figure S1. 1L Treatment Regimens* by Baseline Metastasis and Radiation Claims in 1L at Class Level (A) and Treatment Level (B)

Table S1. ICD-9-CM[23] and ICD-10-CM[24] Codes

Table S2. Baseline Patient Characteristics by Radiation Subgroup

Table S3. All-cause and EC-related HCRU and Conditional Costs by Setting

Table S4. All-cause and EC-related HCRU and Conditional Costs by Setting (Office Clinic Visit, SNF Visit, and Other Medical Visit)

Table S5. All-cause HCRU and Conditional Costs by Metastases at Baseline

Table S6. All-cause and EC-related HCRU and Conditional Costs by Setting Adjusted to 2023

Table S7. All-cause and EC-related HCRU and Conditional Costs by Setting (Office Clinic Visit, SNF Visit, and Other Medical Visit) Adjusted to 2023

Table S8. All-cause HCRU and Conditional Costs by Metastases at Baseline Adjusted to 2023

Table S9. All-cause and EC-related HCRU and Conditional Costs by Setting Adjusted to 2023

This supplementary material has been provided by the authors to give readers additional information about their work.

This is an open-access article distributed under the terms of the Creative Commons Attribution 4.0 International License (CCBY-4.0). View this license's legal deed at http://creativecommons.org/licenses/by/4.0 and legal code at http://creativecommons.org/licenses/by/4.0/legalcode for more information.

Figure S1. 1L Treatment Regimens* by Baseline Metastasis and Radiation Claims in 1L at Class Level (**A**) and Treatment Level (**B**)

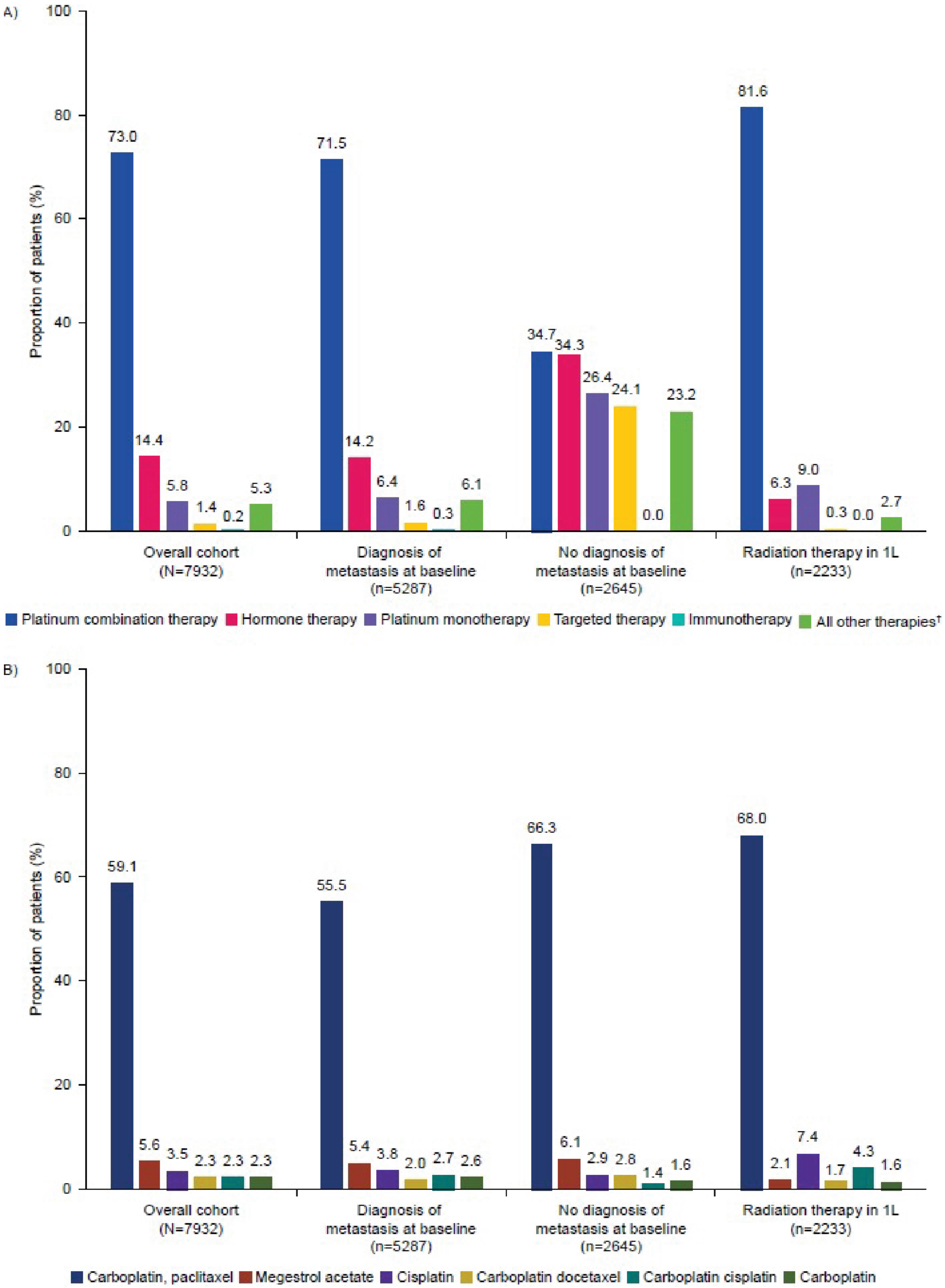

*The full list of anti-cancer therapies assessed in this study is available on request.

†Non-platinum chemotherapies (alone or in combination) recommended for 1L EC in the NCCN Guidelines®, which were not used in combination with platinum chemotherapy, hormone therapy, targeted therapy, or immunotherapy.

Abbreviations: 1L, first-line; EC, endometrial cancer; NCCN, National Comprehensive Cancer Network.

Table S1. ICD-9-CM[23] and ICD-10-CM[24] Codes

Code	Name
**ICD-9-CM 179.x**	Malignant neoplasm of uterus, part unspecified
**ICD-9-CM 182.x**	Malignant neoplasm of body of uterus
**ICD-9-CM 196**	Secondary and unspecified malignant neoplasm of lymph nodes
**ICD-9-CM 197**	Secondary malignant neoplasm of respiratory and digestive systems
**ICD-9-CM 198**	Secondary malignant neoplasm of other specified sites
**ICD-9-CM 199**	Malignant neoplasm without specification of site
**ICD-10-CM C54**	Malignant neoplasm of corpus uteri
**ICD-10-CM C55**	Malignant neoplasm of uterus, part unspecified
**ICD-10-CM C77**	Secondary and unspecified malignant neoplasm of lymph nodes
**ICD-10-CM C78**	Secondary malignant neoplasm of respiratory and digestive systems
**ICD-10-CM C79**	Secondary malignant neoplasm of other and unspecified sites
**ICD-10-CM C80**	Malignant neoplasm, without specification of site
**ICD-10-CM C7B**	Secondary neuroendocrine tumors

Abbreviations: ICD-9-CM, *International Classification of Diseases, Ninth Edition, Clinical Modification*; ICD-10-CM, *International Classification of Diseases, Tenth Edition, Clinical Modification*.

Table S2. Baseline Patient Characteristics by Radiation Subgroup

Patient Characteristics	Radiation Therapy in 1L (N = 2233)	No Radiation Therapy in 1L (N = 5699)
**Age at index (y), mean (SD)**	60.9 (9.2)	61.0 (10.5)
**US Census region, n (%)**		
Midwest	590 (26.4)	1438 (25.2)
Northeast	492 (22.0)	1199 (21.0)
South	757 (33.9)	1944 (34.1)
West	318 (14.2)	906 (15.9)
NR	76 (3.4)	212 (3.7)
**Noncancer CCI, mean (SD)**	3.83 (3.14)	5.01 (3.11)
**No. of noncancer CCI conditions, n (%)**		
0	1487 (66.6)	3342 (58.6)
1–2	622 (27.9)	1825 (32.0)
≥3	124 (5.6)	532 (9.3)
**Payor, n (%)**		
Commercial	1670 (74.8)	4160 (73.0)
Medicare	563 (25.2)	1539 (27.0)
**History of EC surgery, n (%)[a]**		
No EC surgery	239 (10.7)	1561 (27.4)
Subtotal hysterectomy	13 (0.6)	81 (1.4)
Total hysterectomy	1534 (68.7)	2904 (51.0)
Radical hysterectomy	861 (38.6)	1445 (25.4)
Salpingo-oophorectomy	755 (33.8)	1663 (29.2)
Bilateral salpingo-oophorectomy	117 (5.2)	762 (13.4)
Hysterectomy, NOS	550 (24.6)	1342 (23.5)
**Radiation therapy in 1L, n (%)[a]**		
EBRT	819 (36.7)	0 (0.0)
Brachytherapy	1768 (79.2)	0 (0.0)

[a]Surgical categories are not mutually exclusive.
Abbreviations: 1L, first-line; CCI, Charlson Comorbidity Index; EBRT, external beam radiotherapy; EC, endometrial cancer; NOS, not otherwise specified; NR, not reported.

Table S3. All-cause and EC-related HCRU and Conditional Costs by Setting

Setting	All-cause		EC-related	
	**Patients, n (% of Total)**	**Total payment PPPM in USD, median (IQR)**	**Patients, n (% of Total)**	**Total payment PPPM in USD, median (IQR)**
**Non-pharmacy medical encounter**	7925 (99.9)	4605 (2169–9335)	6568 (82.8)	2427 (846–5956)
ED visit	2699 (34.0)	306 (110–814)	408 (5.1)	473 (177–1096)
Inpatient hospitalization	2135 (26.9)	3661 (1732–8747)	1010 (12.7)	4320 (2041–9729)
Outpatient visit	7252 (91.4)	2449 (883–5658)	5453 (68.7)	1561 (464–4059)
**Medication fill**	7932 (100.0)	1446 (435–3951)	7932(100.0)	138 (34–533)
**Total monthly cost**	**7932 (100.0)**	**7696 (3855–14,339)**	**7932 (100.0)**	**2446 (605–6400)**
Metastasis at baseline	5287 (66.7)	8865 (4567–16,470)	5287 (66.7)	2726 (688–7156)
No metastasis at baseline	2645 (33.3)	5717 (2779–10,502)	2645 (33.3)	1943 (476–5121)

See **Table S9** for costs adjusted to 2023.
Abbreviations: EC, endometrial cancer; ED, emergency department; HCRU, healthcare resource utilization; PPPM, per patient per month; USD, US dollars.

Table S4. All-cause and EC-related HCRU and Conditional Costs by Setting (Office Clinic Visit, SNF Visit, and Other Medical Visit)

Setting[a]	All-cause			EC-related		
	**Patients, n (% of Total)**	**Total Payment PPPM in USD, Mean (SD)**	**Total Payment PPPM in USD, Median (IQR)**	**Patients, n (% of Total)**	**Total Payment PPPM in USD, Mean (SD)**	**Total Payment PPPM in USD, Median (IQR)**
**Office clinic visit**	7268 (91.6)	1087 (2068)	411 (114–1205)	4792 (60.4)	930 (1795)	293 (52–1006)
**SNF visit**	134 (1.7)	3717 (10,304)	1993 (778–4346)	59 (0.7)	5454 (15,187)	2575 (1170–5374)
**Other medical visit**	4301 (54.2)	536 (1650)	92 (20–409)	1805 (22.8)	472 (1807)	48 (12–271)

[a]Additional non-pharmacy medical encounters not listed in **Table 2**. See **Table S7** for costs adjusted to 2023.
Abbreviations: EC, endometrial cancer; HCRU, healthcare resource utilization; IQR, interquartile range; PPPM, per patient per month; SNF, skilled nursing facility; USD, US dollars.

Table S5. All-cause HCRU and Conditional Costs by Metastases at Baseline

Setting	Metastasis at Baseline			No Metastasis at Baseline		
	**N (%)**	**Total Payment PPPM in USD, Mean (SD)**	**Total Payment PPPM in USD, Median (IQR)**	**N (%)**	**Total Payment PPPM in USD, Mean (SD)**	**Total Payment PPPM in USD, Median (IQR)**
**Medication fill**	5287 (100.0)	3779 (6533)	1648 (491–4405)	2645 (100.0)	2747 (5192)	1114 (347–3207)
**Non-pharmacy medical encounter**	5282 (99.9)	9553 (13,686)	5378 (2589–10,996)	2643 (99.9)	5737 (8265)	3417 (1598–6824)
ED visit	1982 (37.5)	883 (1881)	324 (114–861)	717 (27.1)	749 (2006)	256 (99–665)
Inpatient hospitalization	1680 (31.8)	9210 (15,854)	4310 (1931–9846)	455 (17.2)	5387 (11,612)	2385 (1181–4721)
Outpatient visit	4900 (92.7)	5226 (8229)	2628 (982–6111)	2352 (88.9)	3893 (5538)	2100 (686–4772)

See **Table S8** for costs adjusted to 2023.
Abbreviations: ED, emergency department; HCRU, healthcare resource utilization; IQR, interquartile range; PPPM, per patient per month; USD, US dollars.

Table S6. All-cause and EC-related HCRU and Conditional Costs by Setting Adjusted to 2023

Setting	All-cause		EC-related	
	**Patients, n (% of Total)**	**Total Payment PPPM in USD, Mean (SD)**	**Patients, n (% of Total)**	**Total Payment PPPM in USD, Mean (SD)**
**Non-pharmacy medical encounter**	7925 (99.9)	8752 (12,982)	6568 (82.8)	5544 (10,154)
ED visit	2699 (34.0)	895 (2024)	408 (5.1)	1016 (1494)
Inpatient hospitalization	2135(26.9)	8875 (15,993)	1010 (12.7)	9974 (1,741)
Outpatient visit	7252 (91.4)	5066 (7916)	5453 (68.7)	3666 (6174)
**Medication fill**	7932 (100.0)	3631 (6488)	7932 (100.0)	1143 (3879)
**Total monthly cost**	**7932 (100.0)**	12,375 (14,831)	**7932 (100.0)**	5734 (10,225)
Metastasis at baseline	5287 (66.7)	14,083 (16,191)	5287 (66.7)	6493 (11,583)
No metastasis at baseline	2645 (33.3)	8963 (10,873)	2645 (33.3)	4215 (6473)

Abbreviations: EC, endometrial cancer; ED, emergency department; HCRU, healthcare resource utilization; IQR, interquartile range; PPPM, per patient per month; USD, US dollars.

Table S7. All-cause and EC-related HCRU and Conditional Costs by Setting (Office Clinic Visit, SNF Visit, and Other Medical Visit) Adjusted to 2023

Setting[a]	All-cause			EC-related		
	**Patients, n (% of Total)**	**Total Payment PPPM in USD, Mean (SD)**	**Total Payment PPPM in USD, Median (IQR)**	**Patients, n (% of Total)**	**Total Payment PPPM in USD, Mean (SD)**	**Total Payment PPPM in USD, Median (IQR)**
**Office clinic visit**	7268 (91.6)	1149 (2186)	434 (121–1274)	4792 (60.4)	983 (1897)	310 (55–1063)
**SNF visit**	134 (1.7)	3929 (10,892)	2107 (822–4594)	59 (0.7)	5765 (16,053)	2722 (1237–5680)
**Other medical visit**	4301 (54.2)	567 (1744)	97 (21–432)	1805 (22.8)	499 (1910)	51 (13–286)

[a]Additional non-pharmacy medical encounters not listed in **Table 2**.
Abbreviations: EC, endometrial cancer; HCRU, healthcare resource utilization; IQR, interquartile range; PPPM, per patient per month; SNF, skilled nursing facility; USD, US dollars.

Table S8. All-cause HCRU and Conditional Costs by Metastases at Baseline Adjusted to 2023

Setting	Metastasis at Baseline			No Metastasis at Baseline		
	**N (%)**	**Total Payment PPPM in USD, Mean (SD)**	**Total Payment PPPM in USD, Median (IQR)**	**N (%)**	**Total Payment PPPM in USD, Mean (SD)**	**Total Payment PPPM in USD, Median (IQR)**
**Medication fill**	5287 (100.0)	3994 (6906)	1742 (519–4656)	2645 (100.0)	2904 (5488)	1178 (367–3390)
**Non-pharmacy medical encounter**	5282 (99.9)	10,098 (14,466)	5685 (2737–11,623)	2643 (99.9)	6064 (8736)	3612 (1689–7213)
ED visit	1982 (37.5)	933 (1988)	342 (121–910)	717 (27.1)	792 (2120)	271 (105–703)
Inpatient hospitalization	1680 (31.8)	9735 (16,758)	4556 (2041–10,407)	455 (17.2)	5694 (12,274)	2521 (1248–4990)
Outpatient visit	4900 (92.7)	5524 (8698)	2778 (1038–6459)	2352 (88.9)	4115 (5854)	2220 (725–5044)

Abbreviations: EC, endometrial cancer; ED, emergency department; HCRU, healthcare resource utilization; IQR, interquartile range; PPPM, per patient per month; USD, US dollars.

Table S9. All-cause and EC-related HCRU and Conditional Costs by Setting Adjusted to 2023

Setting	All-cause		EC-related	
	**Patients, n (% of Total)**	**Total Payment PPPM in USD, Median (IQR)**	**Patients, n (% of Total)**	**Total Payment PPPM in USD, Median (IQR)**
**Non-pharmacy medical encounter**	7925 (99.9)	4868 (2293–9867)	6568 (82.8)	2565 (894–6296)
ED visit	2699 (34.0)	323 (116–860)	408 (5.1)	500 (187–1158)
Inpatient hospitalization	2135 (26.9)	3870 (1831–9246)	1010 (12.7)	4566 (2157–10,284)
Outpatient visit	7252 (91.4)	2589 (933–5981)	5453 (68.7)	1650 (490–4290)
**Medication fill**	7932 (100.0)	1528 (460–4176)	7932 (100.0)	146 (36–563)
**Total monthly cost**	**7932 (100.0)**	8135 (4075–15,157)	**7932 (100.0)**	2585 (639–6765)
Metastasis at baseline	5287 (66.7)	9371 (4827–17,409)	5287 (66.7)	2881 (727–7564)
No metastasis at baseline	2645 (33.3)	6043 (2937–11,101)	2645 (33.3)	2054 (503–5413)

Abbreviations: EC, endometrial cancer; ED, emergency department; HCRU, healthcare resource utilization; IQR, interquartile range; PPPM, per patient per month; USD, US dollars.
